# Genomic Imprinting: A New Epigenetic Perspective of Sleep Regulation

**DOI:** 10.1371/journal.pgen.1006004

**Published:** 2016-05-26

**Authors:** Valter Tucci

**Affiliations:** Neuroscience and Brain Technologies Department, Istituto Italiano di Tecnologia (IIT), Genova, Italy; Geisel School of Medicine at Dartmouth, UNITED STATES

## Overview

A growing corpus of data indicates that epigenetic mechanisms regulate sleep and sleep-wake cycles. Here, I discuss recent evidence showing that genomic imprinting, an epigenetic mechanism that regulates parent-of-origin effects in mammals, is involved in the control of rapid eye movement (REM) sleep. REM sleep is an evolutionarily recent form of sleep that is characterized by important electrophysiological, metabolic, and thermoregulatory changes. The link between imprinting and REM sleep offers new insights into the epigenetic mechanisms underlying sleep physiology.

Sleep is associated with significant changes in the expression of many genes, which suggests that sleep regulates a number of physiological and/or behavioural functions. Approximately 15% of transcripts across the genome oscillate along sleep-wake cycles, more than 40% of protein-coding genes oscillate in at least one tissue [1], and a number of molecular pathways sense epigenetic changes that depend on sleep. For example, sleep loss disrupts the circadian rhythm in 20% of the oscillating genes in the brain [2] and affects the DNA binding of clock genes by acting on the methylation state of their promoters [3]. Moreover, significant methylation changes have recently been reported in mice when their sleep-wake cycles are manipulated starting in the early stages of development after birth [4].

There are various theories for the function of sleep. For example, one theory was proposed that the function of sleep is to decrease energy demands, while another theory suggested that sleep is to restore cellular and subcellular processes [5]. At the moment, a new theory proposes that a positive selective pressure for sleep is associated with the role of sleep in fundamental mechanisms that regulate the communication between neurons [6] and, more generally, in connectivity processes that wire the brain [7]. Neuronal properties are the most frequently investigated functions of sleep, and these studies indicate that sleep has a significant role in cognitive processes (e.g., memory consolidation [8]).

Novel insights are now emerging for the epigenetic mechanisms regulating sleep. In particular, the role of genomic imprinting in sleep regulation has been systematically studied in recent years. Genomic imprinting is an epigenetic mechanism that results in the allele-specific expression of approximately 200 genes according to the parental origin and is unique to mammals among vertebrates [9]. Imprinted genes play a crucial role in the placenta and prenatal development and, after birth, have been demonstrated to control important metabolic and physiological functions (e.g., thermogenesis) as well as behavioural and cognitive processes [10,11]. Imprinted genes have important roles during the perinatal period [11], which is a crucial time window in development, for the formation and integration of all biological systems, including the homeostatic control of sleep and the formation of the internal (circadian) clock [12,13].Recent studies have reported that imprinted genes are involved in important control mechanisms for both the ‘S” (homeostatic) and “C” (circadian) processes of sleep (see Box 1).

**Box 1. Sleep Regulation**

Sleep results from the synergism between at least two major processes: a homeostatic (process S) regulatory mechanism that depends on the accumulation of the sleep drive during wakefulness, and a circadian (process C) self-sustained mechanism that sets the time for sleeping and waking throughout the 24-hour daily cycle [14]. Central and peripheral oscillators control the circadian process, and the main light-dependent oscillator is composed of a dense group of neurons in the suprachiasmatic nucleus (SCN) of the hypothalamus [15]. However, the sleep homeostatic process is controlled by a neurobiological network that is primarily distributed in the brain [16] and involves several (and perhaps local) brain mechanisms [17]. Moreover, sleep is commonly divided into two major physiological stages: rapid eye movement (REM) sleep and non-REM (NREM) sleep. During REM sleep, there is an increase in neuronal and metabolic activities, a reduction in muscle tone, and several irregularities in autonomic and thermoregulatory functions [18], whereas NREM sleep is significantly more quiet. REM sleep apparently contravenes the restorative aspects of sleep; however, the function of this “paradoxical” state remains unknown. Although REM sleep may serve important functions, a lack of REM sleep (e.g., the severe suppression of REM sleep caused by specific pharmacological treatments [19]) has no major consequences for survival in humans; however, severe detrimental effects have been observed in rats [5].

For example, opposite imprinting defects at chromosome 15q11–13 are responsible for opposite sleep phenotypes as well as opposite neurodevelopmental abnormalities, namely the Prader-Willi syndrome (PWS) and the Angelman syndrome (AS) [14,20]. Whilst the PWS is due to loss of paternal expression of alleles, the AS is due to loss of maternal expression. The 15q11–13 region consists of several genes that are biparentally expressed (i.e., GABA receptor genes), paternally expressed (i.e., *MKRN3*, *MAGEL2*, *NECDIN*, and a C/D small nucleolar RNA [snoRNA] cluster), and maternally expressed (i.e., *UBE3A*, a HECT-domain E3 ubiquitin ligase [also known as E6-AP] that is involved in proteasome degradation). Maternal additions or paternal deletions of alleles at chromosome 15q11–13 are characterized by temperature control abnormalities, excessive sleepiness, and specific sleep architecture changes, particularly REM sleep deficits [21–23]. Conversely, paternal additions or maternal deletions at chromosome 15q11–13 are characterized by reductions in sleep and frequent and prolonged night wakings [24,25].

In the mouse, specific neurodevelopmental processes, including sleep and the circadian clock, are associated with the maternally expressed *Ube3a*. Ehlen et al. [26] explored the circadian and sleep functions in *Ube3a*^*m-/p+*^ mice, and they report that the *Ube3a* gene is an important player in the regulation of sleep homeostasis (the process “S” of sleep). In particular, the architecture of non-REM and REM sleep in *Ube3a*^*m-/p+*^ mice differed from that in wild-type mice; however, although the overall total amount of non-REM sleep over a 24-hour period was similar among the two genotypes, the mice carrying the maternal deletion of the *Ube3a* gene displayed a ~20% reduction in REM sleep compared with the control.

Studying the process “C” of sleep, conflicting data have emerged regarding whether *Ube3a* controls the circadian clock. The maternal deletion of the *Ube3A* allele in the *Ube3a*^*m-/p+*^ mouse line has led to significant [27] and nonsignificant [26] differences in the length of the circadian period. However, Shi and colleagues [27] demonstrated a role for *Ube3a* in regulating the turnover of a core element of the circadian transcriptional-translational feedback loop, *Bmal1*. This work is consistent with the recent finding that *Bmal1* is a target of E6-AP in the ubiquitination process [28]. Moreover, although the paternal *Ube3a* allele is considered silenced throughout the brain, Ehlen et al. [26] demonstrated that the protein is widely expressed in the SCN of *Ube3a*^*m-/p+*^ mice. Therefore, in contrast to the rest of the brain, *Ube3a* appears biallelically expressed in the SCN.

Within the same 15q11–13 region, we reported that the paternally expressed noncoding snoRNA, *SNORD116*, has an important role in sleep physiology and thermoregulation [29]. In particular, we observed that the deletion of *SNORD116*/*Snord116* in humans and mice led to increased REM sleep and REM intrusions during wakefulness. The regulation of REM sleep is strongly influenced by daily variations of thermoregulatory demand [30]. In our study, mice lacking *Snord116* displayed an increased peripheral body temperature, which suggests that *Snord116* may regulate the interplay between sleep and daily thermoregulation profiles. However, the circadian rhythm of the behavioural activity in these mice was not altered, which indicated that the *Snord116* locus regulates metabolic-dependent sleep homeostasis but not circadian rhythms in adulthood, though targeting a different gene of the central chromosome 7 suggested light-dependent circadian control. Indeed, the paternally expressed protein-coding gene *Magel2* was found to modulate light-dependent circadian rhythms [31]. An additional example of the genomic imprinting modulation of sleep was shown in our previous work on the maternally expressed imprinted gene *Gnas* [32], which maps to the distal imprinted region of mouse chromosome 2. The loss of *Gnas* imprinting dramatically reduced REM sleep and was associated with an increase in the core body temperature in mice [32].

Changes in body temperature and REM sleep in the *Snord116* and *Gnas* models represent different situations in terms of expressed gene dosage. Loss of expression of paternal *Snord116* results in enhanced REM sleep, implying that the normal function of this gene is to decrease REM sleep. The effect on body temperature suggests that a normal function of paternal *Snord116* is to decrease peripheral body temperature. Double expression of maternal *Gnas* (due to loss of imprinting) results in decreased REM sleep, which might imply that the normal role of imprinted single dose *Gnas* is also to decrease REM (assuming additive effects of expressed gene dosage of *Gnas* on REM). The effect on body temperature suggests that a normal function of maternal *Gnas* is to elevate core body temperature. Reduced body temperature during sleep is caused by heat dissipation, which increases the skin temperature and promotes heat dissipation from the core to the periphery [33].

Genomic imprinting in endothermic animals is involved in specific functions that counteract hypothermia. Several imprinted genes (i.e., *Gnas*, *Gnasxl*, *Ndn*, *Dlk1*, and *Dio3*) that are expressed in brown adipocytes control the thermogenesis process through the mitochondrial uncoupling protein 1 (*Ucp1*) [10,32]. We showed that genomic imprinting alterations of *Gnas* affect *Ucp1* expression, temperature control, and sleep [32].

In conclusion, alterations in the core-to-peripheral temperature gradient, such as those determined by genomic imprinting defects, determine physiological disruption of normal sleep. Taken together, these results indicate that both maternal and paternal imprinted genes significantly control REM sleep, which may occur through the control of circadian variations of thermoregulation. Covariation between ecological, physiological, and phylogenetic factors can account for significant interactions between genomic imprinting, sleep, and body temperature (see Box 2).

**Box 2. Genomic Imprinting and REM Sleep Evolution**

**Fig 1:**
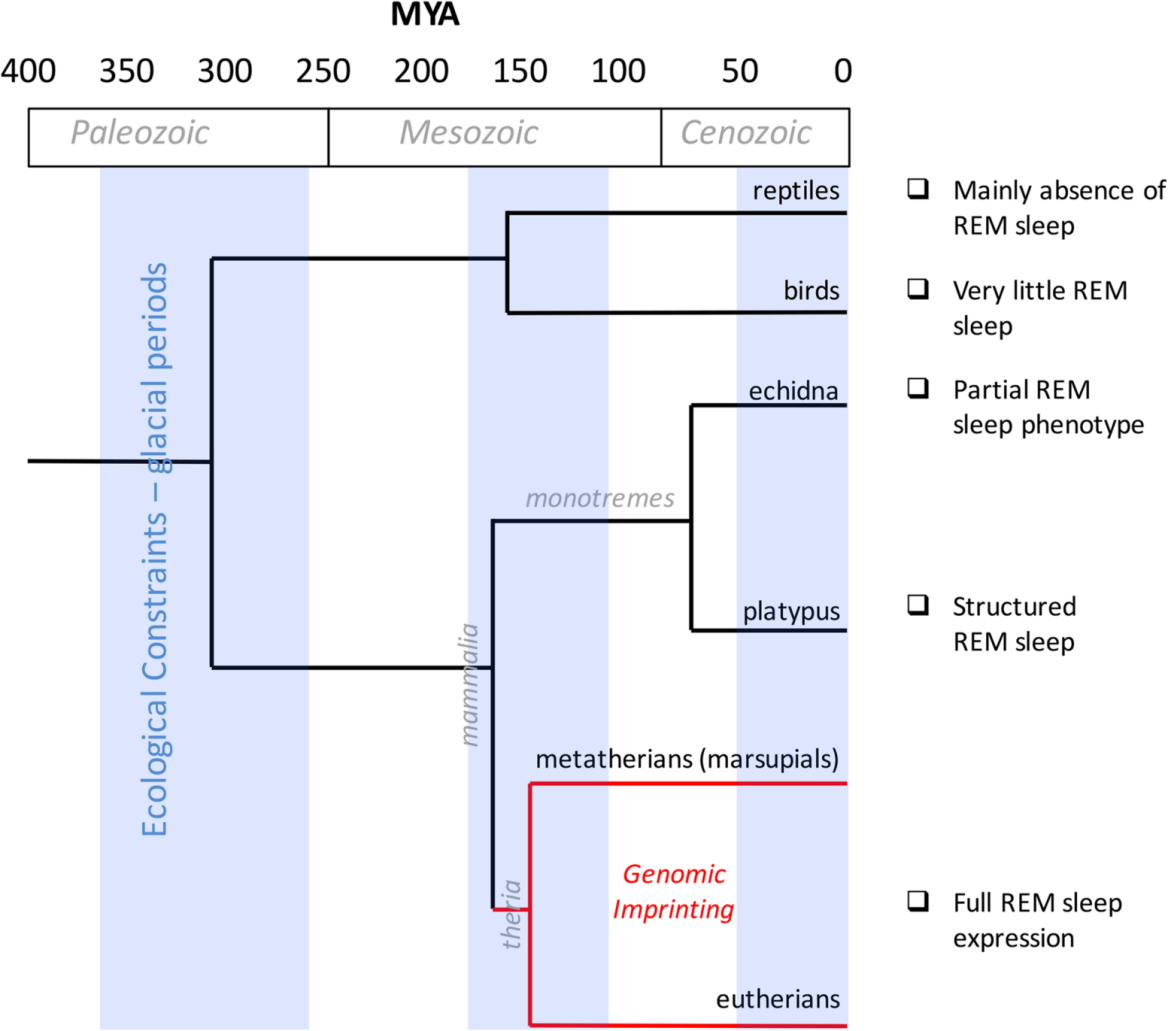
Evolution of REM sleep and genomic imprinting. The timeline (expressed as millions of years ago, MYA) on the horizontal axis maps geological and glacial periods and the evolutionary divergence that occurred among reptiles, birds, and mammals (monotremes, eutherians, and marsupials). The red line delineates the presence of genomic imprinting in therians. A description of REM sleep according to different species is annotated on the right.

Genomic imprinting evolved in therians (Fig 1) and is significantly associated with placentation [34]. A primitive form of placentation originated approximately 150 million years ago, during the divergence between placental mammals and egg-laying monotremes [34]. At that time, the epigenetic marks of imprinting appeared in certain genes, and then the early placental lineage between marsupials and eutherians divided. Interestingly, a monoallelic imprinting mechanism is also observed in marsupials, although this mechanism evolved separately and resulted in several differences between the imprinted genes of eutherians and marsupials [35].

The decreased thermoregulation during REM sleep may be related to the thermoregulation that occurs in reptiles. However, a number of studies focusing on the sleep cycles of reptiles and amphibians have failed to identify REM sleep [36,37] or showed little evidence of REM sleep [38,39]. The REM-like sleep patterns that occur in mammals and in a reduced form in birds [40] suggest that this form of sleep evolved independently within the two clades (Fig 1) [41]. Several predictors of the presence of REM sleep across phylogenetic orders have been proposed. For example: the animal's body size, whether it is a prey species, whether it is exposed to unsafe sleeping situations, and whether, soon after birth, the young requires nourishment from the mother (altricial species) or are able to survive on their own (precocial species). Although phylogenetic studies have presented contradictory results with regard to these predictors, in studies of placental and marsupial mammals, all of the predictors of the full aspects of REM sleep have been confirmed [41]. The echidna presents REM-like neuronal activity discharge [41] but lacks the classical behavioural, electrophysiological, and metabolic features of REM sleep [42,43], and these traits supported the initial assertion that REM is absent in monotremes.

All species evolved within specific climatic niches [44,45], or a range of temperature and environmental conditions at which species-specific metabolic processes developed. These ecological niches are crucial factors for determining specific traits (e.g., behavioural, physiological, and metabolic traits). In mammals, monotremes are characterized by the lowest body temperatures, whereas marsupials and eutherians are characterized by the highest body temperatures. Moreover, for specific increments in body mass, mammalian body temperatures have been reported to increase, while avian body temperatures have been reported to decrease, which indicates positive and negative scaling, respectively [46]. It is reasonable to speculate that the link between sleep and imprinting developed during the evolutionary process of adaptive radiation, in which speciation occurs because of ecological opportunities. In particular, the evolution of REM sleep physiology may have been biologically consistent with genomic imprinting within a rapidly divergent endothermic lineage (Fig 1).

The investigation of genomic imprinting effects in sleep has produced intriguing results that suggest important connections between imprinting and sleep in evolutionary processes. However, more imprinted genes need to be investigated. Genomic imprinting may serve as a novel experimental and theoretical model to assess the function of sleep; nevertheless, there is insufficient information at the moment to interpret the actual results into different theories about the origin of imprinting.

The “genomic imprinting hypothesis of sleep” remains in its infancy, and several aspects require attention and further investigation. For example, we recently demonstrated that parent-of-origin effects are important in homeostatic responses to sleep loss [47]. However, if genomic imprinting, which provides a monoallelic mechanism for a small subset of genes, is important for sleep, then it must be clarified why specific parent-of-origin regulatory processes are required rather than a random allelic inactivation process (see Box 3).

**Box 3. Parent-of-Origin Effects and Sleep**

The homeostatic process of sleep, which is classically triggered by sleep deprivation, is measured as a rebound effect of electrophysiological parameters and gene expression changes following sleep loss. By studying the reciprocal crosses of two mouse strains that differ in their homeostatic response to sleep deprivation (the AKR/J and DBA/2J lines), we observed differences in gene expression [47]. AKR/J mice show a significant rebound after six hours of sleep deprivation, whereas DBA/2J mice show only a mild response following sleep deprivation. Following sleep deprivation, AKR/J mice display a higher rebound in core circadian clock genes, including *Bmal1*, *Clock*, *Cry1*, *Cry2*, *Per1*, and *Per2*, relative to DBA/2J mice. Interestingly, we observed a different sleep rebound level in reciprocal heterozygous F1 mice, and certain clock genes were differentially expressed between the two F1 cohorts. To date, nine differentially regulated genes have been identified in AKR/JxDBA/2J sleep-deprived F1 mice and seven differentially regulated genes have been identified in DBA/2JxAKR/J sleep-deprived F1r mice. In this investigation, we identified specific upstream mechanisms of regulation involving signalling pathways (i.e., DICER1, PKA), growth factors (CSF3 and BDNF), and transcriptional regulators (EGR2 and ELK4) that were modulated by parental effects.

Sleep is the most substantial state during development (i.e., it occupies two-thirds of the day in newborns) and plays a fundamental role in developmental processes; furthermore, genomic imprinting is crucial for growth, development, and neurogenesis [10,48]. Therefore, investigations focusing on the interplay between sleep and specific developmental genomic imprinting mechanisms may reveal important new avenues for investigating the neurodevelopmental mechanisms of sleep.
